# Hybrid capture shotgun sequencing detected unexpected viruses in the cerebrospinal fluid of children with acute meningitis and encephalitis

**DOI:** 10.1007/s10096-024-04795-x

**Published:** 2024-03-04

**Authors:** Cristian Launes, Juan Camacho, Marina Pons-Espinal, F. Xavier López-Labrador, Cristina Esteva, María Cabrerizo, María Dolores Fernández-García, Marta Fogeda, Josefa Masa-Calles, Noemí López-Perea, Juan Emilio Echevarría, Carmen Muñoz-Almagro, David Tarragó

**Affiliations:** 1 Institut de Recerca Sant Joan de Déu, Hospital Sant Joan de Déu, Barcelona, Spain; 2 CIBER Epidemiology and Public Health (CIBERESP), Madrid, Spain; 3 Departament de Medicina i Especialitats Medicoquirúrgiques, Facultat de Medicina i Ciències de la Salut, Universitat de Barcelona, Barcelona, Spain; 4 Centro Nacional de Microbiología, Instituto de Salud Carlos III, Majadahonda- Pozuelo km 2, 28220 Majadahonda, Spain; 5 Genomics & Health Department, FISABIO-Public Health Foundation, Valencia, Spain; 6 Department of Microbiology and Ecology, Medical School, University of Valencia, Valencia, Spain; 7 Hologic Iberia SL, Madrid, Spain; 8 Centro Nacional de Epidemiología, Instituto de Salud Carlos III, Avda Monforte de Lemos 5, Madrid, Spain; 9 Department of Medicine, Universitat Internacional de Catalunya, Barcelona, Spain

**Keywords:** Cerebrospinal fluid (CSF), Undiagnosed viral meningoencephalitis, Paediatric meningitis and/or encephalitis (M/ME), Viral metagenomics, Human herpesvirus 7 (HHV-7), BK virus, Epstein-Barr virus (EBV), Human endogenous retrovirus K-113 (HERV-K113)

## Abstract

**Purpose:**

Investigation of undiagnosed cases of infectious neurological diseases, especially in the paediatric population, remains a challenge. This study aimed to enhance understanding of viruses in CSF from children with clinically diagnosed meningitis and/or encephalitis (M/ME) of unknown aetiology using shotgun sequencing enhanced by hybrid capture (HCSS).

**Methods:**

A single-centre prospective study was conducted at Sant Joan de Déu University Hospital, Barcelona, involving 40 M/ME episodes of unknown aetiology, recruited from May 2021 to July 2022. All participants had previously tested negative with the FilmArray Meningitis/Encephalitis Panel.

HCSS was used to detect viral nucleic acid in the patients’ CSF. Sequencing was performed on Illumina NovaSeq platform. Raw sequence data were analysed using CZ ID metagenomics and PikaVirus bioinformatics pipelines.

**Results:**

Forty episodes of M/ME of unknown aetiology in 39 children were analysed by HCSS. A significant viral detection in 30 CSF samples was obtained, including six parechovirus A, three enterovirus ACD, four polyomavirus 5, three HHV-7, two BKV, one HSV-1, one VZV, two CMV, one EBV, one influenza A virus, one rhinovirus, and 13 HERV-K113 detections. Of these, one sample with BKV, three with HHV-7, one with EBV, and all HERV-K113 were confirmed by specific PCR. The requirement for Intensive Care Unit admission was associated with HCSS detections.

**Conclusion:**

This study highlights HCSS as a powerful tool for the investigation of undiagnosed cases of M/ME. Data generated must be carefully analysed and reasonable precautions must be taken before establishing association of clinical features with unexpected or novel virus findings.

**Supplementary Information:**

The online version contains supplementary material available at 10.1007/s10096-024-04795-x.

## Introduction

Infectious diseases of the central nervous system (CNS), including meningitis, encephalitis, and meningoencephalitis, carry a high disease burden. These typically acute infections present significant morbidity and mortality, especially in children [1]. Rapid diagnosis is crucial for these medical emergencies to guide early and appropriate therapy. Unfortunately, microbiological confirmation and laboratory diagnosis remain challenging for most of these infections. Current diagnostic workflows typically rely on differential diagnosis based on patient history, clinical presentation, and imaging findings, followed by serial targeted testing methods like PCR assays for specific pathogens. Yet, these methods are clearly inefficient, as the aetiological agent of paediatric meningitis/encephalitis (M/ME) remains unidentified in up to 40–60% of patients [2, 3]. Rapid syndromic molecular arrays based on multiplex PCR tests which include the most prevalent microorganisms causing M/ME have been developed. For instance, EliTech commercializes CNS infection panels for detecting seven prevalent virus causing M/ME (namely, Meningitis Viral ELITe MGB® Panel for HSV1, HSV2, and VZV; Meningitis Viral 2 ELITe MGB Panel for enterovirus, parechovirus and adenovirus; and EliTech HPeV RT-PCR Test for parechovirus (types 1–6)) and BioFire Diagnostics commercializes the FilmArray Meningitis/Encephalitis Panel [4], which detects 14 pathogens, seven of them virus: cytomegalovirus (CMV), enterovirus (EV), herpes simplex virus type 1 (HSV-1), herpes simplex virus 2 (HSV-2), human herpes virus type 6 (HHV-6), human parechovirus and varicella- zoster virus (VZV). These panels, along with in-house multiplex real-time PCR developed at reference laboratories and used elsewhere [5], are valuable for diagnosing their target pathogens. Nevertheless, broader nucleic acid amplification tests for a wider range of potential pathogens in CSF are needed, as existing tools are limited to detecting known viruses or predefined panels of viral targets [4, 6–8]. The emergence and re-emergence of numerous clinically significant viruses causing CNS infections, not included in these panels and PCRs, highlight the need for novel or unexpected viral pathogen identification [9].

Next-Generation Sequencing (NGS) technology is transforming microorganism detection and characterization. Unlike the low throughput of Sanger sequencing, NGS is a high throughput technology that sequences multiple DNA molecules in parallel, allowing the sequencing of hundreds of millions of DNA molecules at once. A significant advantage of metagenomic NGS over targeted methods is its unbiased sequencing of all nucleic acid in a clinical sample, enabling the detection of both unexpected and expected pathogens. Additionally, metagenomic NGS can detect and quantify minor subpopulations of a specific pathogen. Incorporating pan-viral hybrid capture assays to enrich sequences covering all known families of human and animal viral pathogens in the workflow addresses the primary disadvantage of lower sensitivity compared to real-time PCR [10, 11]. These approaches necessitate further clinical interpretation by virologists, clinicians, and bioinformaticians.

This study aims to enhance understanding of viruses in the CSF of paediatric patients with clinically diagnosed M/ME of unknown aetiology using viral HCSS. As NGS technologies for pathogen identification continue to evolve, determining optimal approaches for patient-care scenarios where tests are likely to be conducted becomes crucial.

## Patients and methods

### Patients and clinical samples

Prospective recruitment of patients aged ≥ 3 months and ≤ 14 years, who were treated for M/ME of unknown aetiology at the Sant Joan de Déu University Hospital (Barcelona), a renowned centre in paediatrics, was conducted from May 2021 to July 2022. All patients had previously tested negative in CSF using routine microbiological methods (bacterial cultures and FilmArray ME Panel (Biomérieux, Marcy-l'Étoile, France)). The FilmArray ME Panel tests for specific DNA/RNA fragments of: *Escherichia coli* K1, *Haemophilus influenzae*, *Listeria monocytogenes*, *Neisseria meningitidis*, *Streptococcus agalactiae*, *Streptococcus pneumoniae*, CMV, enterovirus, HSV-1, HSV-2, HHV-6, human parechovirus, VZV and *Cryptococcus neoformans/gattii*.

Inclusion criteria were as follows: patients whose parents or guardians agreed to participate in the study under informed consent and had available surplus of CSF samples (at least 200 μl). Diagnostic criteria for meningitis included fever, headache, neck stiffness or bulging fontanelle, with or without altered mental status and pleocytosis in CSF (CSF White Cell Count > 5/mm3) [12]. Encephalitis diagnostic criteria included altered mental status (defined as decreased or altered level of consciousness, lethargy, or personality change) lasting ≥ 24 h with no alternative identified cause (major criteria) and at least two minor criteria (documented fever ≥ 38 °C within 72 h before or after presentation; generalized or partial seizures not fully attributable to a pre-existing seizure disorder; new onset of focal neurologic findings; CSF WBC count ≥ 5/cubic mm; abnormality of brain parenchyma on neuroimaging suggestive of encephalitis that is either new from prior studies or appears acute in onset; abnormality on electroencephalography consistent with encephalitis and not attributable to another cause) [13]. Exclusion criteria included lack of informed consent or inability to obtain the minimum surplus volume of CSF sample for HCSS analysis.

### Shotgun sequencing

Sequencing was performed using a pan-viral (DNA and RNA viruses) metagenomic approach as previously reported [5, 14].

#### Sample processing

Total nucleic acid was extracted from 200 µl of cerebrospinal fluid using the QiAmp Mini Elute Virus Spin Kit (Qiagen, Hilden, Germany) with no RNA carrier, eluted in 30 µl nuclease-free water, aliquoted and stored at -80 °C until further processing. One microlitre was used to quantify RNA using the QuantiFluor® RNA System (Promega, Madison, WI, USA). A CSF sample previously positive for enterovirus by PCR was processed through all steps and served as a positive control. Four negative controls (HyClone™ HyPure Molecular Biology Grade Water) representing each round of extraction procedure were used. These controls underwent the entire shotgun sequencing protocol described herein.

#### Library preparation

Previous studies carried out in our laboratory [5, 14] indicated that active infections caused by DNA virus were detectable through an RNA shotgun sequencing protocol. This approach allows for the identification of viral gene expression and the precise determination of the viral pathogen responsible for the active infection.

Libraries were constructed using the NEBNext® Ultra II Directional RNA Library Prep Kit for Illumina® (New England Biolabs, Ipswich, MA, USA), following the manufacturer’s protocol. The initial amount of RNA was 1 ng/μl in a final volume of 5 μl for the CSF samples; the fragmentation time was 13 min at 94 °C, the dilution of the NEBNext-adaptor was 1:25; and the number of cycles in the amplification step was 13. In addition to the above-described RNA Library Prep Kit, NEBNext Multiplex Oligos for Illumina®- Index Primer Set 1 (New England Biolabs, Ipswich, MA, USA) and AMPure XP SPRI Reagent by Beckman Coulter (Life Sciences Division Headquarters, Indianapolis, IN, USA) were used to complete the library construction. Nucleic acid was quantified by QuantiFluor® dsDNA System (Promega, Madison, WI, USA), and quality (integrity and size of libraries) was verified by Bioanalyzer High Sensitivity DNA Analysis System (Agilent Technologies, Inc., Santa Clara, CA, USA). Samples underwent further processing when they met the manufacturer’s recommended criteria in terms of quality (300–500 base pairs) and quantity (at least 1 ng/μl).

#### Viral nucleic acid enrichment by hybrid capture

Twist Target Enrichment Standard Hybridization v2 (Twist Bioscience, South San Francisco, CA, USA) was used following the manufacturer’s protocol. The Twist Target Enrichment protocol was used to generate viral-enriched DNA libraries for sequencing on Illumina next-generation sequencing (NGS) systems. This method is based on hybridization probes and covers reference sequences for 3,153 viruses, including 15,488 different strains. Individual libraries were combined by equal mass (33.33 ng per library) into six capture enrichment pools. Library pools were processed at high drying speed using the Savant SpeedVac DNA130 Vacuum Concentrator (Thermo Fisher Scientific, Waltham, MA, USA). Then, the library pools were resuspended and hybridized with the custom biotin-labelled probe panel for a minimum of 16 h, and then hybrids were captured using streptavidin according to the manufacturer’s protocol. In the amplification step, the maximum number of cycles (× 15) allowed by the protocol was used.

#### Sequencing

DNA from hybrid capture libraries was quantified with the QuantiFluor dsDNA System (Promega Madison, WI, USA) and quality verified for sequencing by the Bioanalyzer High Sensitivity DNA Analysis System (Agilent Technologies, Inc. Santa Clara, CA, USA). Shotgun sequencing of the captured libraries was performed on a NovaSeq 6000 Illumina platform (Illumina Inc., San Diego, CA, USA) using NovaSeq 6000 Standard S2 Reagent Kit v1.5 (300 cycles, with 150 cycles per strand in a paired-end format).

### Data analysis and identification of virus

Sequencing raw data were processed and analysed using the Chan-Zuckerberg ID (CZ ID) metagenomics pipeline (https://czid.org/). We applied a background model using four negative controls. Viral discovery was performed with the PikaVirus pipeline (developed at the Bioinformatics Unit, National Centre for Microbiology, Spain, https://github.com/BU-ISCIII/PikaVirus). In both pipelines, the analysis involved multiple steps, including quality control, removal of human and non-relevant sequences, assembly of viral genomes or contigs, and the identification of viral sequences through sequence alignment and comparison to known viral databases.

### Viral PCR confirmation

Viral pathogens detected by shotgun sequencing were confirmed using specific PCR tests, when available. Enterovirus and parechovirus were confirmed through multiplex real-time PCR as previously described by Cabrerizo et al. [15]. Similarly, multiplex real-time PCR was used for EBV, CMV, and HHV-7 as previously described by Recio et al. [16]. The detection of HERV-K113 was confirmed via a PCR assay as detailed by Moyes et al. [17]. For rotavirus, PCR methods described by Mijatovic-Rustempasic et al. [18], along with the Allplex™ GI-Virus Assay (Seegene Inc., Seoul, Souht Korea) were utilised. Influenza A virus was identified by PCR as described by Ruiz-Carrascoso et al. [19]. HSV-1 and VZV were confirmed through multiplex RT real-time PCR as previously described by Castellot et al. [5]. Lastly, the presence of human polyomavirus (BKV and JCV) was established by a real-time PCR assay as described by Bárcena-Panero et al. [20].

### Statistical analysis

Comparisons of categorical data were conducted using the Pearson chi-square test or the Fisher exact test. For continuous variables that were not normally distributed, the Mann–Whitney U-test analysis was used. A p-value of less than 0.05 was considered statistically significant. Statistical analysis was performed with SPSS v22.0 software (IBM Corp, Armonk, NY, USA).

## Results

### Patients’ main characteristics

During the study, 48 out of 55 episodes of M/ME had no aetiological diagnosis following routine clinical tests. Out of these, 40 had sufficient CSF sample for NGS analysis and were included in the study. One patient developed two different episodes. The median age of the patients was 3.6 years (IQR: 1.3–6.6), with 25 (62%) being male. Twenty (50%) had a previously known condition, primarily oncologic-haematologic diseases, autistic spectrum disorders, and other chronic neurological disabilities. One patient was tested with two different samples; therefore 41 samples were analysed.

### Hybrid capture shotgun sequencing

The forty-one CSF samples, along with one enterovirus-positive CSF used as a control, and four negative controls were shotgun sequenced and processed using the CZ ID metagenomics pipeline v8.2. Average reads per sample were 43.47 million, with an average of 13.18 million reads passing filters (host and low quality) per sample. Significant parameter detection was achieved in 22 CSF samples using four negative controls in a background model computed on the CZ ID pipeline. A Z-score of 100 indicates that the virus was not detected in negative controls. HERV-K113 was detected in 13 CSF samples by the PikaVirus pipeline, of which 9 showed co-detection with other viruses. It is noteworthy that the CZ ID pipeline did not detect HER-K113 in any sample, presumably because this virus is considered a part of the human genome. Significant results from the CZ ID metagenomics pipeline v8.2 are shown in Table 1. Virus detection was further confirmed by specific PCR in three HHV-7, one BKV, and one EBV cases.

**Table 1:** Significant results and metagenomic data following background model generation in the CZ ID metagenomics pipeline

ID	Virus	Score	Z-score	rPM	r	Contig	Contig r	%id	L	E-value	#PCR	*K113
+ C	Enterovirus B	2,303,065,968	100.0	115,153.3	398,406	4	397,819	88.2	2,265.2	10^–30^	ND	-
43	Human betaherpesvirus 5 (CMV)	2,474,149	99.0	252.4	9,982	1	9,982	99.6	276.0	10^–139^	-	+
34	Human alphaherpesvirus 1 (HSV-1)	15,157,281	100.0	761.7	25,498	0	0	99.4	149.9	10^–88^	ND	-
33	Parechovirus A	107,885,513	100.0	4,232.7	29,46	0	0	98.1	150.7	10^–86^	-	-
33	Human betaherpesvirus 7 (HHV-7)	92,809,701	100.0	4,639.4	32,291	0	0	99.2	131.9	10^–76^	+	-
32	Influenza A virus	1,428,827	100.0	125.4	4,172	0	0	99.2	119.5	10^–68^	-	-
31	Parechovirus A	6,496,194	100.0	325.0	14,828	0	0	98.0	150.9	10^–86^	-	-
30	Human betaherpesvirus 7 (HHV-7)	29,704,988	100.0	1,485.2	13,908	0	0	99.3	130.1	10^–75^	+	-
29	Human polyomavirus 5	114,071,724	100.0	5,703.6	57,147	1	50,76	99.7	211.5	10^–109^	ND	-
27	Parechovirus A	18,677,430	100.0	933.9	50,914	1	50,914	98.7	300.0	10–148	-	+
26	Human polyomavirus 5	17,798,496	100.0	889.9	15,88	0	0	99.5	150.4	10–89	ND	-
26	Human betaherpesvirus 7 (HHV-7)	59,758,900	100.0	2,987.9	53,317	0	0	99.2	150.9	10–89	+	+
24	BK virus	37,394,477	100.0	1,869.8	64,824	0	0	99.2	150.8	10–89	ND	-
23	Parechovirus A	3,265,663	100.0	128.1	3,135	0	0	98.4	150.7	10–87	-	+
22	Human alphaherpesvirus 3 (VZV)	140,950,516	100.0	7,048.7	230,761	0	0	99.0	79.4	10–40	ND	-
20	Human betaherpesvirus 5 (CMV)	11,400,851	99.0	575.9	13,15	0	0	99.2	79.7	10–41	-	-
19	Rhinovirus A	57,095,753	100.0	5,709.6	211,874	1	211,874	99.1	231.0	10–113	-	-
17	Parechovirus A	21,878,060	100.0	1,093.9	29,502	1	29,5	98.7	299.5	10–147	-	-
15	Enterovirus C	67,715,364	100.0	3,385.8	106,414	0	0	98.0	150.3	10–86	-	+
14	Human polyomavirus 5	10,004,214	100.0	500.2	19,844	0	0	99.3	151.0	10–89	ND	-
12	Enterovirus D	47,923,385	100.0	2,396.2	142,384	2	142,384	98.8	255.0	10–124	-	-
8	Parechovirus A	92,610,318	100.0	3,326.9	76,779	0	0	97.7	149.6	10–85	-	-
7	Human polyomavirus 5	48,593,615	100.0	2,429.7	26,45	0	0	99.3	137.9	10–80	ND	-
6	BK virus	2,073,413	100.0	103.7	7,704	0	0	98.9	150.5	10–88	+	-
6	Human gammaherpesvirus 4 (EBV)	54,016,950	100.0	2,700.8	200,68	2	200,544	100.0	243.7	10–121	+	-
2	Enterovirus A	301,782,140	100.0	15,089.3	102,692	0	0	97.7	149.0	10–84	-	-

*+ C Positive control; ND* Not determined; *ID* Identification of the clinical sample; *#PCR* Viral PCR confirmation; *K113 HERV-K113 positive PCR; *Virus* Best matching virus found; *Score* Experimental ranking score used to prioritize microbes based on their abundance within the sample (rPM) and their comparison to control samples (Z-score); *Z Score* Statistic used to evaluate the prevalence of microbes in the sample as compared to background contaminants; *rPM* Number of reads aligning to the taxon in the NCBI NR/NT database, per million reads sequenced; *Contig* Number of assembled contigs aligning to the taxon; *Contig r* Total number of reads across all assembled contigs; *%id* Average percent-identity of alignments to NCBI NT/NR; *L* Average length of the local alignment for all contigs and reads assigned to this taxon; *E value* Average expect value (e-value) of alignments to NCBI NT/NR. Chan Zuckerberg ID—Detect & Track Infectious Diseases (czid.org)

Other viruses were detected in 17 CSF samples. However, these hits were dismissed as feasible results because they were also present in the negative controls, resulting in scores far from 100 according to the CZ ID background model generation. Nonetheless, PCR testing was conducted when available, revealing the detection of human papillomavirus 115 in one CSF sample and additional HERV-K113 detections in four of these samples. Data from these non-relevant detections are shown in Table 2.

**Table 2:** Non-significant results and metagenomic data following background model generation in the CZ ID metagenomics pipeline

ID	Virus	Score	Z-score	rPM	r	contig	contig r	%id	L	E-value	#PCR	*K113
46	Alphatorquevirus	6,617	1.8	2,067.8	24,943	0	0	99.1	95.9	10^–52^	ND	
46	Betapapillomavirus	334	0.7	355.6	4,29	0	0	99.4	150.9	10^–89^	Human papillomavirus 115 confirmed	
44	Betapapillomavirus 3	16,187	1.8	2,609.7	31,38	0	0	98.3	149.9	10–86	ND	+
42	Mastadenovirus	449	1.8	70.2	3,141	0	0	98.5	102.8	10^–55^	ND	
42	Rotavirus	1	−0.3	3,939.8	176,317	0	0	93.6	151.1	10^–77^	negative*	
41	Rotavirus	−311	−0.1	2,287.7	91,494	1	91,494	98.9	274.0	10^–134^	negative*	
40	Betapapillomavirus	97,901	0.7	767.1	10,086	0	0	97.6	127.3	10^–69^	ND	
38	Rotavirus	−101	−0.9	65.2	9,78	0	0	99.1	96.8	10^–52^	negative*	
36	Human coronavirus 229E	49,918	1.8	7,800.1	191,298	1	33,6	99.0	161.6	10^–90^	ND	
34	Human mastadenovirus C	16,177,165	66.9	1,839.6	61,584	0	0	99.2	149.0	10^–87^	ND	+
33	Circovirus	3,184	0.7	6,368.2	44,324	0	0	99.1	54.7	10^–24^	ND	
32	Human mastadenovirus C	2,262,297	45.0	558.5	18,576	1	18,576	99.6	236.0	10^–116^	ND	
31	Betapapillomavirus	19,686	−0.1	630.4	28,765	0	0	98.1	150.4	10^–86^	ND	+
30	Rotavirus	78,037	1.3	24,455.8	229,006	0	0	98.5	140.0	10^–80^	negative*	
26	Rotavirus	3	−0.6	6,419.9	114,558	2	86,296	97.8	197.1	10^–96^	negative*	
23	Rotavirus A	−1,124	−0.8	839.6	20,544	1	12,294	99.2	201.8	10^–105^	negative*	+
21	Rotavirus A	−7,123	−0.6	10,742.2	685,613	0	0	98.4	150.3	10^–87^	negative*	
17	Human mastadenovirus C	26,673,538	91.9	2,086.3	56,265	0	0	98.9	149.6	10^–87^	ND	
16	Rotavirus A	776	0.2	6,271.9	211,39	0	0	98.6	149.4	10^–86^	negative*	

*ND* Not determined; *ID* Identification of the clinical sample; *#PCR* Viral PCR confirmation; *K113 HERV-K113 positive PCR; *Virus* Best matching virus found; *Score* Experimental ranking score used to prioritize microbes based on their abundance within the sample (rPM) and their comparison to control samples (Z-score); *Z Score* Statistic used to evaluate the prevalence of microbes in the sample as compared to background contaminants; *rPM* Number of reads aligning to the taxon in the NCBI NR/NT database, per million reads sequenced; *Contig* Number of assembled contigs aligning to the taxon; *Contig r* Total number of reads across all assembled contigs; *%id* Average percent-identity of alignments to NCBI NT/NR; *L* Average length of the local alignment for all contigs and reads assigned to this taxon; *E value* Average expect value (e-value) of alignments to NCBI NT/NR. Chan Zuckerberg ID—Detect & Track Infectious Diseases (czid.org)

### Clinical characteristics

The main clinical characteristics of the patients are summarized in Table 3. When considering only significant results from the CZ ID HCSS analysis, there were no differences in terms of age, sex, or previously known conditions between individuals with a detection and those without any detection. Similarly, no differences were observed in the main clinical symptoms, CSF characteristics, or blood characteristics. However, patients requiring admission to the PICU had a higher rate of positive CZ ID HCSS results compared to those who did not require pediatric intensive care unit (PICU) admission. The length of hospital stay and the incidence of sequelae were similar between the two groups.

**Table 3:** Main clinical characteristics and differences between patients with and without significant CZ ID HCSS detections

	Total *N* = 40	Significant HCSS results		
		No	Yes	*p*
Age (y-old)	3.6 (1.3–6.6)	4.6 (1.7–6.1)	2.5 (0.9–7.0)	0.51
Sex (male)	25	9/18	16/22	0.14
Chronic conditions	20	10/18	10/22	0.52
Symptoms				
- Fever	26	11/18	15/22	0.89
- Meningism	6	2/18	4/22	0.53
- Irritability	14	8/18	6/22	0.26
- Vomiting	7	1/18	6/22	0.11
- Lethargy	21	11/18	10/22	0.32
- Headache	5	3/18	2/22	0.64
- Tremor	4	3/18	1/22	0.31
- Myoclonus	3	1/18	2/22	1
- Seizures	18	8/18	10/22	0.94
- Ataxia	10	6/18	4/22	0.30
- Paresis	6	2/18	4/22	0.67
- CNP affected	6	1/18	2/22	1
- Loss of sensitivity	1	0/18	1/22	1
CSF				
- Leucocytes (/mm3)	5 (0–20)	5 (3–15)	5 (0–40)	0.92
- Neutrophils (%)	0 (0–20)	0 (0–0)	0 (2–21)	0.16
- Proteins (mg/dL)	22 (16–40)	26 (17–41)	20 (16–41)	0.42
- Neopterin (nmol/L)	64 (47–240)	240 (152–472)	53 (37–117)	0.11
Blood				
-Absolute lymphocyte count (x10E3/mm3)	2.7 (2.0–4.1)	2.3 (1.7–3.1)	2.9 (2.3–4.9)	0.13
- C-reactive protein (mg/dL)	1.6 (0.1–7.1)	3.1 (0.4–7.1)	0.4 (0–0.7)	0.29
Altered MRI	15/28	7/12	8/16	0.66
PICU	14/40	3/18	11/22	**0.03**
LOS	7 (4–17)	7 (4–13)	7 (4–25)	0.59
Persistent symptoms at day 14 after admission	17	6/18	11/22	0.29

*CNP* Cranial nerves pairs; *CSF* Cerebrospinal fluid; *LOS* Length-of-stay; *MRI* Magnetic resonance imaging; *PICU* Pediatric intensive care unit

The specific clinical characteristics associated with each patient with HCSS detections was obtained are summarized in Table S1 of the supplementary information file.

## Discussion

HCSS proved to be a successful approach for detecting both RNA and DNA viruses in CSF from children with meningoencephalitis of unknown aetiology. Compared to the standard-of-care screening using the FilmArray Meningitis/Encephalitis (FA/ME) panel, HCSS achieved additional significant viral detection in 30 cases, some of which were not included in the FA/ME panel. These detections included six cases of parechovirus A, three of enterovirus ACD, three of HHV-7, two of BK virus, four of polyomavirus 5, one of HSV-1, one of VZV, two of CMV, one of EBV, one of influenza A virus, and one of rhinovirus by using CZ ID pipeline. In addition, 13 detections of HERV-K-113 by using Pika Virus pipeline were found. Of all these, one sample with BKV, three with HHV-7, one with EBV, and all with HERV-K-113 were further confirmed by PCR.

The rare positive identifications through HCSS, in the absence of specific PCR confirmation, posed a challenge for explanation. We introduced negative controls at the outset of each round of nucleic acid extraction as a standard procedure to contamination control. These negative controls underwent the same processing steps as our experimental samples, from nucleic acid extraction to sequencing. Containing no target DNA or RNA, they served as a baseline for detecting any contamination that might occur during sample handling, processing and due to kits, reagents and instrumentation. Furthermore, the CZ ID pipeline expressed the normalized results of each sample in terms of reads per million (rPM), Z and Z-score metrics, which were highly valuable for interpreting results and making them readily comparable to data generated across the scientific community. Moreover, the Z-score metric in the CZ ID sample report is based on the prevalence of each virus in the selected negative control samples, with which the CZ ID pipeline built the background model. It used rPM as the metric of relative abundance and rPM values were normalized for sequencing depth. In analysing results from a particular sample, the Z-score metric can be used to provide insight into whether a particular virus was present in the negative control samples. A virus present at a higher abundance in the sample than in the controls will have a Z-score > 1. If a virus is not found in the set of control samples, the Z-score is set to 100, and if the virus is not found in the sample but is present in the controls, the Z-score is set to -100.

Regarding herpesviruses, establishing HHV-7, EBV, and CMV as the cause of encephalitis is often challenging because viral nucleic acids can also be detected during latency and asymptomatic viral reactivation. Interestingly, three cases of HHV-7 were detected by shotgun sequencing and further confirmed by PCR, with co-detection of parechovirus A and human polyomavirus 5 in two cases, respectively Ideally, a cohort of healthy children would assist with interpreting results, but obtaining cerebrospinal fluid from healthy children is legally challenging. Using patient groups with non-infectious diseases may lead to misinterpretations, since herpesviruses are known to reactivate during inflammatory processes. Since parechovirus A is a common cause of neurological disease in young children, it is reasonable to consider this virus as the cause of encephalitis, even though only HHV-7 was further confirmed by PCR in this CSF. Most neurological diagnostics rely on molecular tests for specific pathogens, making it difficult to identify multi-viral infections. However, when a metagenomics approach was used, some co-infections were observed [20]. Single case reports and case series have previously described HHV-7 -related encephalitis or encephalopathy in both immunocompetent and immunocompromised children and adults [21, 22]. The detection of HHV-7 DNA by shotgun sequencing, further confirmed by PCR in the CSF of the patient with neurological disease, alongside the exclusion of all alternative aetiological causes (in accordance with the clinical practice guidelines of the Infectious Diseases Society of America [23]), supports HHV-7 as a possible cause of the encephalitis. This is based on the criteria established by Schwartz et al. [24]. Nevertheless, HHV-7 is highly prevalent and has been detected in normal brain tissue [25]. Clinical judgment is crucial in determining the clinical significance of HHV-7 detection in the CSF. In a multicentre prospective surveillance study of viral agents causing meningoencephalitis, HHV-7 and EBV were found in 10% and 6% of cases, respectively [26]. Again, the role of EBV as a cause of CNS disease must be deduced from its clinical setting.

We detected one case of HSV-1 and another of VZV, which could not be confirmed by PCR due to insufficient clinical samples and were not previously detected with the FA/ME test. A recent study conducting diagnostic test accuracy meta-analysis (including sensitivity and subgroup analyses) reported suboptimal sensitivities for HSV-1, concluding that the FA/ME test is excellent for ruling in but limited for ruling out CNS infections [27]. Nonetheless, the patient improved with only 48 h of specific treatment and showed no typical radiological signs of herpetic encephalitis. We also detected CMV, which is one of the most clinically recognized viral pathogens in immunocompromised patients, in two cases not confirmed by specific PCR, possibly due to low viral load as suggested by the low rPM parameter from the CZ ID pipeline. For genomic detection of low virus concentrations, HCSS has proven more sensitive compared to untargeted shotgun sequencing [28].

Regarding enterovirus and parechovirus, we detected three and six cases, respectively. The species Parechovirus A currently consists of 19 different human parechovirus types (HPeV) 1 to 19. Parechovirus A and enteroviruses (EV) are common viral causes of meningoencephalitis in children [29, 30]. However, the FA-ME assay failed to detect them in the CSF samples, possibly due to the lower sensitivity of PCR compared to HCSS or because PCR primers used in the initial screening lacked sensitivity and capacity to amplify EV sequences of all known genotypes [31]. The recently available EliTech HPeV RT-PCR Test [6] increases HPeV detection chances, being able to detect up to six different types. However, reduced sensitivity due to reagent competition, and lack of flexibility to modify the panel are major obstacles for the applications of multiplex CNS pathogen detection panels in clinical laboratories [32].

Regarding polyomavirus, we detected BKV and Merkel-cell polyomavirus (MCPyV; Human polyomavirus 5) in two and four samples, respectively. BKV is common in kidney disease of immunocompromised patients but rarely reported in neurological infection. However, in a study of 2,062 CSF samples from neurological patients, BKV was detected by specific PCR in 20 patients diagnosed with progressive multifocal leukoencephalopathy or multiple sclerosis [33]. MCPyV infection is usually asymptomatic, but it can cause life-threatening pathologies such as Merkel cell carcinoma in immunocompromised hosts. MCPyV was detected in 22 neurological patients with acute ME by PCR elsewhere, but likely as a bystander rather than as an aetiological agent [34].

The presence and confirmation of HERV-K113 by specific PCR is relevant. HERVs are genetic elements in the human genome that originated from ancient retroviral germline infections [35]. Of note, HERV- K113 is the only endogenous retrovirus known to produce viral infective particles [36] and is associated with certain autoimmune diseases [17]. The clinical implications of this retrovirus in numerous pathologies are still debated, but a study by Moyes et al. [17] found its prevalence significantly higher in multiple sclerosis and Sjögren's syndrome.

Rhinovirus is a common respiratory pathogen in children year-round; however, its CNS involvement is extremely rare, with few reported cases. Current literature does not definitively identify this virus as a causative pathogen. Furthermore, it has been associated with cerebellitis [37], a condition absents in our case. Although rhinovirus is not typically tested in cerebrospinal fluid (CSF), it belongs to the Enteroviridae family. Most PCR tests cannot distinguish it from other enteroviruses. Nervous system injury associated with influenza is a leading cause of influenza-related child deaths, with fatality rates up to 30% [38]. Neurological symptoms of brain injury typically appear on the same day or within days after cold symptom onset, commonly involving convulsions and consciousness alterations. Common types of nervous system injury associated with influenza include influenza-associated encephalopathy (IAE) [39], Reye’s syndrome, Guillain-Barré syndrome, haemorrhagic shock encephalopathy syndrome [40], and acute necrotizing encephalopathy (ANE) [39], with ANE being the most severe [41]. Our case, not linked to respiratory symptoms, had uncertain relevance.

As discussed, using a background model in the CZ ID pipeline facilitated results interpretation, distinguishing contamination or artifacts. Human coronavirus HCoV-NL63, HCoV-229E, HCoV-HKU1, and HCoV-OC43 account for 15%–30% of common cold cases [42]. Human coronavirus 229E causes common colds and self-limiting respiratory infections. However, the aetiologic role of HCoVs in neurological diseases remains unproven [43]. Anelloviruses, such as Alphatorquevirus (TTV), are ubiquitous in humans and are nearly constantly shed by infants [44], indicating early-life infections. TTV is recognized as the main component of the human viral flora [45]. While they are considered non-pathogenic, Anelloviridae might be associated with the occurrence of some disorders, including respiratory disorders in childhood [46]. Though exceptional Torque teno virus cases have been described in the literature [47], it is usually considered a contaminant or bioinformatics artifact. Few laboratory reagents appear to be entirely free from contamination, particularly by ssDNA viruses, and predominantly circoviruses [48–53].

This study’s findings may have significant implications for patient management, treatment strategies, and public health interventions. However, limitations include the necessity of meticulous sample collection, storage, and processing to enhance the accuracy of viral pathogen detection. Enrichment procedures, like the hybrid capture used here, can increase the percentage of viral nucleic acid relative to host nucleic acid. Yet, the choice of method greatly affects results. For instance, depleting methylated DNA to eliminate human DNA can also inadvertently remove viral DNA from viruses that rely on human machinery, such as Epstein-Barr virus (EBV). Utilising appropriate positive and negative controls is crucial for adjusting the assay threshold and evaluating the plausibility of identified pathogens. The methodology used in this study enabled detecting expected and unexpected pathogens, requiring further clinical interpretation by virologists, bioinformaticians, and the judgment of experienced physicians.

### Supplementary Information

Below is the link to the electronic supplementary material.

Supplementary file1 (DOCX 31.2 KB)

## Data Availability

All data generated and/or analysed during the current study are included in this manuscript and its Supporting Information files.
