# Supplementary material for: Hybrid capture shotgun sequencing detected unexpected viruses in the cerebrospinal fluid of children with acute meningitis and encephalitis
Source: Eur J Clin Microbiol Infect Dis. 2024 Mar 4;43(5):863–73. doi: 10.1007/s10096-024-04795-x (PMC11108891; doi:10.1007/s10096-024-04795-x)
Supplement: Supplementary file 1 — Supplementary file1 (DOCX 31.2 KB) [file 10096_2024_4795_MOESM1_ESM.docx]

**Table S1.** Overview of the main characteristics and outcomes in children with HCSS detections.

| **ID** | **HCSS CZ ID detection** | **Age-range** | **Comorbidity** | **Symptoms** | **PICU admission** | **CSF WCC (/mm3)** | **CSF protein (g/L)** | **MRI findings** | **Other relevant information** | **Persistent symptoms at day 14** |
| --- | --- | --- | --- | --- | --- | --- | --- | --- | --- | --- |
| 2 | Enterovirus A | 2-4 y | Autism | Vomits, lethargy, abdominal distension | Yes | 5 | 15 | - | Suspected abdominal occlusion | No |
| 12 | Enterovirus D | 3m-1y | No | Altered consciousness, myoclonus | No | 0 | 16 | Normal | Enterovirus detection by PCR in stool | No |
| 15 | Enterovirus C | 2-4y | Oncologic malignancy | Fever, altered consciousness, seizures | Yes | 0 | 13 | Normal | - | No |
| 34 | Human alphaherpesvirus 1 | 3m–1y | Oncologic malignancy | Fever, altered consciousness, seizures | Yes | 0 | 16 | Normal | Received acyclovir for 48 hours | No |
| 22 | Human alphaherpesvirus 3 (VZV) | 5-9y | No | Fever, altered consciousness, seizures | No | 50 | 17 | Normal | Received acyclovir for 48 hours | No |
| 20 | Human betaherpesvirus 5 (CMV) | 2-4y | No | Fever, altered consciousness, tremor, seizures, gastrointestinal | No | 5 | 12 | Normal | Received acyclovir for 48 hours | No |
| 43 | Human betaherpesvirus 5 (CMV) | 10-14y | No | Fever, paresis, gastrointestinal | No | 28 | 74 | Bilateral punctate subcortical lesions in T2/FLAIR | - | Yes |
| 26 | Human betaherpesvirus 7  Polyomavirus 5 | 2-4y | No | Fever, altered consciousness, paresis | No | 62 | 22 | Neuritis in high spinal nerve roots and cervical myelitis | - | Yes |
| 30 | Human betaherpesvirus 7 | 5-9y | No | Dysautonomic crisis, paresis, seizures | Yes | 1 | 40 | Diffuse cortico-subcortical T2/FLAIR hyperintensities | - | Yes |
| 6 | Human gammaherpesvirus 4 (EBV)  Polyomavirus 1 | 3m-1y | No | Fever, altered consciousness, paresis | No | 27 | 23 | Diffuse cortico-subcortical T2/FLAIR hyperintensities | Received acyclovir for 48 hours | Yes |
| 32 | Influenza A | 3m-1y | Preterm | Fever, altered consciousness, seizures | Yes | 68 | 300 | - | No respiratory symptoms | No |
| 8 | Parechovirus A | 5-9y | Autism | Altered consciousness, seizures | Yes | 0 | 18 | Normal | - | Yes |
| 17 | Parechovirus A | 2-4y | No | Altered consciousness, ataxia, seizures, gastrointestinal | No | 0 | 14 | - | Adenovirus detection in stools | No |
| 23 | Parechovirus A | 3m-1y | No | Fever, meningism | No | 130 | 68 | - | - | No |
| 27 | Parechovirus A | 5-9y | Chromosomal disorder | Altered consciousness, ataxia, seizures, gastrointestinal | No | 36 | 45 | Normal | - | No |
| 31 | Parechovirus A | 10-14y | Autism | Fever, altered consciousness, seizures | Yes | 6 | 26 | Focal cortico-subcortical T2/FLAIR hyperintensity | - | Yes |
| 33 | Parechovirus A  Human betaherpesvirus 7 | 3m-1y | No | Fever, altered consciousness, sepsis-like | Yes | 0 | 11 | - | Suspected bacterial superinfection with C-reactive protein and procalcitonin elevation | No |
| 24 | Polyomavirus 1 | 5-9y | No | Fever, meningism, ataxia, vomits | No | 150 | 40 | Diffuse cortico-subcortical T2/FLAIR hyperintensities | - | Yes |
| 7 | Polyomavirus 5 | 10-14y | Admission due to an episode of altered consciousness at infancy | Fever, meningism, seizures, vomiting | No | 19 | 50 | Focal cortico-subcortical T2/FLAIR hyperintensity | - | No |
| 14 | Polyomavirus 5 | 3m-1y | Epileptic encephalopathy | Fever, altered consciousness, seizures | Yes | 0 | 16 | Normal | - | Yes |
| 29 | Polyomavirus 5 | 3m-1y | Congenital heart defect | Fever, altered consciousness, seizures | Yes | 0 | 20 | - | - | Yes |
| 19 | Rhinovirus | 5-9y | No | Altered consciousness, impaired vision | No | 19 | 31 | Diffuse cortico-subcortical T2/FLAIR hyperintensities and optical neuritis | Anti-MOG + | Yes |

Co-detections: ^*^polyomavirus 1 and other human herpesvirus, ^**^ other human herpesvirus with parechovirus A, ^ⴕ^ polyomavirus 5 and other human herpesvirus

CSF: cerebrospinal fluid; m: month; PCR: Polymerase chain reaction; WCC: white-cell count; y: year
